# The First Reported Case of Metoprolol-Induced Pemphigus Foliaceus in the United States: A Critical Report and Review of Literature

**DOI:** 10.7759/cureus.9203

**Published:** 2020-07-15

**Authors:** Tomer Lagziel, Margarita Ramos, Grace F Rozycki, Charles S Hultman, Mohammed Asif

**Affiliations:** 1 Plastic Surgery, Johns Hopkins University School of Medicine, Baltimore, USA; 2 Medicine, Tel-Aviv University, Sackler School of Medicine, Tel-Aviv, ISR

**Keywords:** pemphigus, autoimmune, dermatology, infection, metoprolol

## Abstract

Pemphigus is a rare family of autoimmune disorders characterized by epithelial and mucosal blisters. Pemphigus foliaceus (PF) commonly affects the scalp, face, and trunk. Lesions often arise as superficial blisters and develop into scaly, crusted erosions. Management includes corticosteroids with immunosuppressants. Novel therapies include immunoadsorption and active clinical trials. We present the only reported case of metoprolol-induced PF in the United States (US), with an extremely complicated hospital course.

A 66-year-old male patient with a history of hypertension, diabetes, and hyperlipidemia presented to his doctor with a blistering, pruritic rash that started after switching to metoprolol for hypertension treatment.

PF is very rare in North America. Given its solely superficial penetration, it creates no direct fatal complication. However, the developing blisters and subsequent wounds are susceptible to a wide array of secondary infections, which can be life-threatening.

## Introduction

Pemphigus is a family of devastating blistering conditions that are characterized by disrupted keratinocyte to keratinocyte adhesion which forms blisters in the epithelium of the skin and mucous membranes [1]. The prevalence of pemphigus in the United States (US) is 0.005% of overall adults [2]. One subtype is pemphigus foliaceus (PF) that only affects the cutaneous layer (sparing mucosal membrane) and includes subcorneal acantholytic blisters [3]. PF most commonly affects the scalp, face, and trunk. The lesions contributing to this condition usually begin presenting as superficial blisters but eventually develop into scaly and crusted erosions. During the course of development of the condition, the skin lesions can either remain local or spread to cover larger areas of the body [4].

As it is an autoimmune condition, the factors that lead to or cause PF are not well understood. It is generally accepted that both genetic and environmental factors contribute to the development of pemphigoid diseases. It has also been recorded that certain drugs, thiols in particular, can induce PF [2,5]

However, PF due to treatment with metoprolol has never been reported in the US, as is seen with our case report.

In cases where PF is environmentally triggered or drug induced, the trigger should be determined and removed from contact with the patient. Medical intervention for pemphigus includes corticosteroids in combination with steroid-sparing immunosuppressant drugs, such as azathropine, mycophenolate, and cyclosprin [3,4,6]. 

Surgical intervention is usually not necessary for isolated-cutaneous lesions and usually only implemented in severe mucosal invasion [7].

## Case presentation

**Figure 1:**
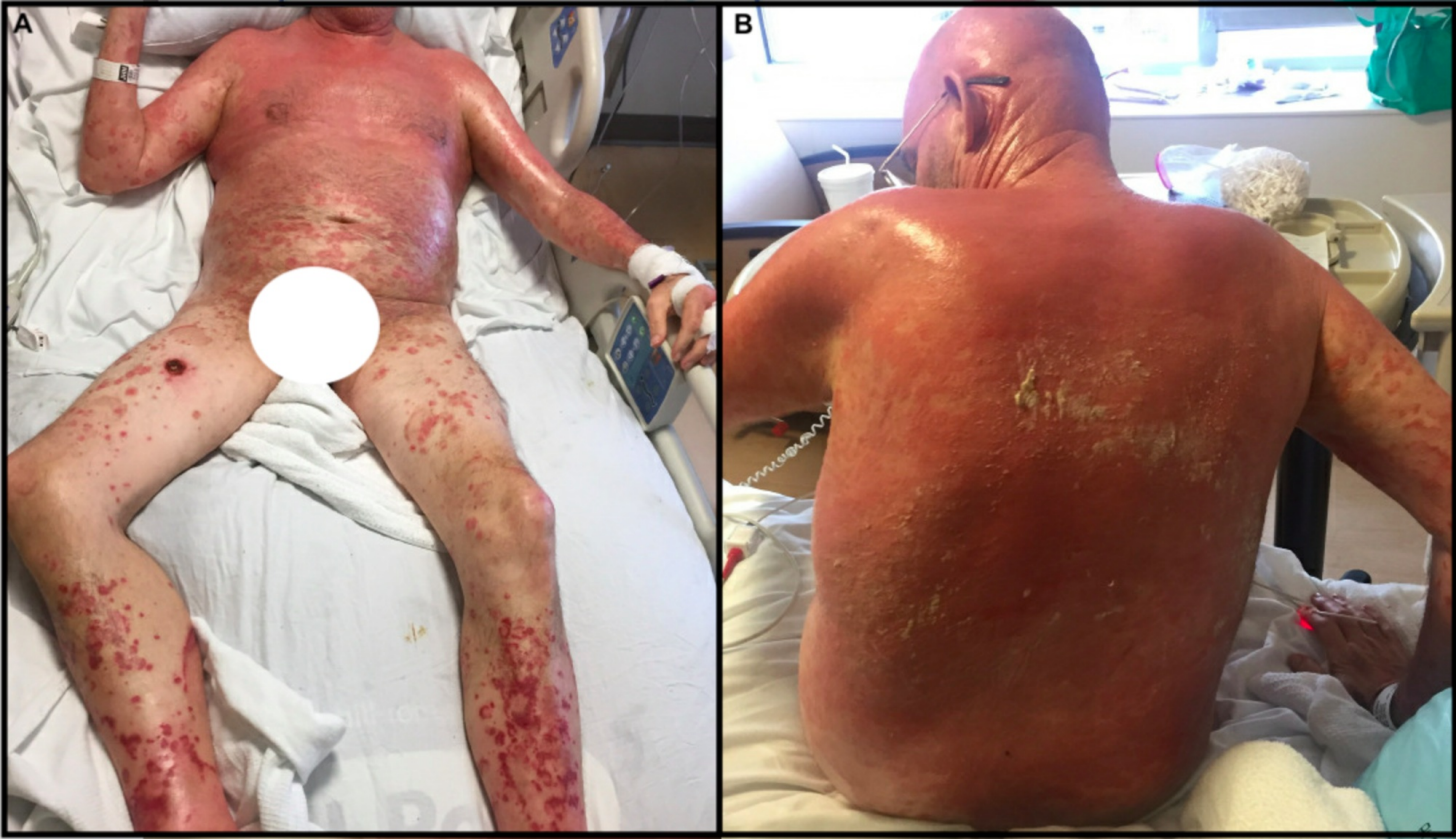
Initial Presentation with Pemphigus Foliaceus. (A – Anterior Torso, B – Posterior Torso)

A 66-year-old male patient with a medical history of hypertension, diabetes, and hyperlipidemia presented to his primary care physician with a blistering, pruritic rash that started after switching to metoprolol from atenolol for treatment of uncontrolled hypertension. The rash became infected with superimposed *Staphylococcus aureus *and when symptoms worsened he was sent to the emergency department (ED) for evaluation (Figure 1). 

**Figure 2:**
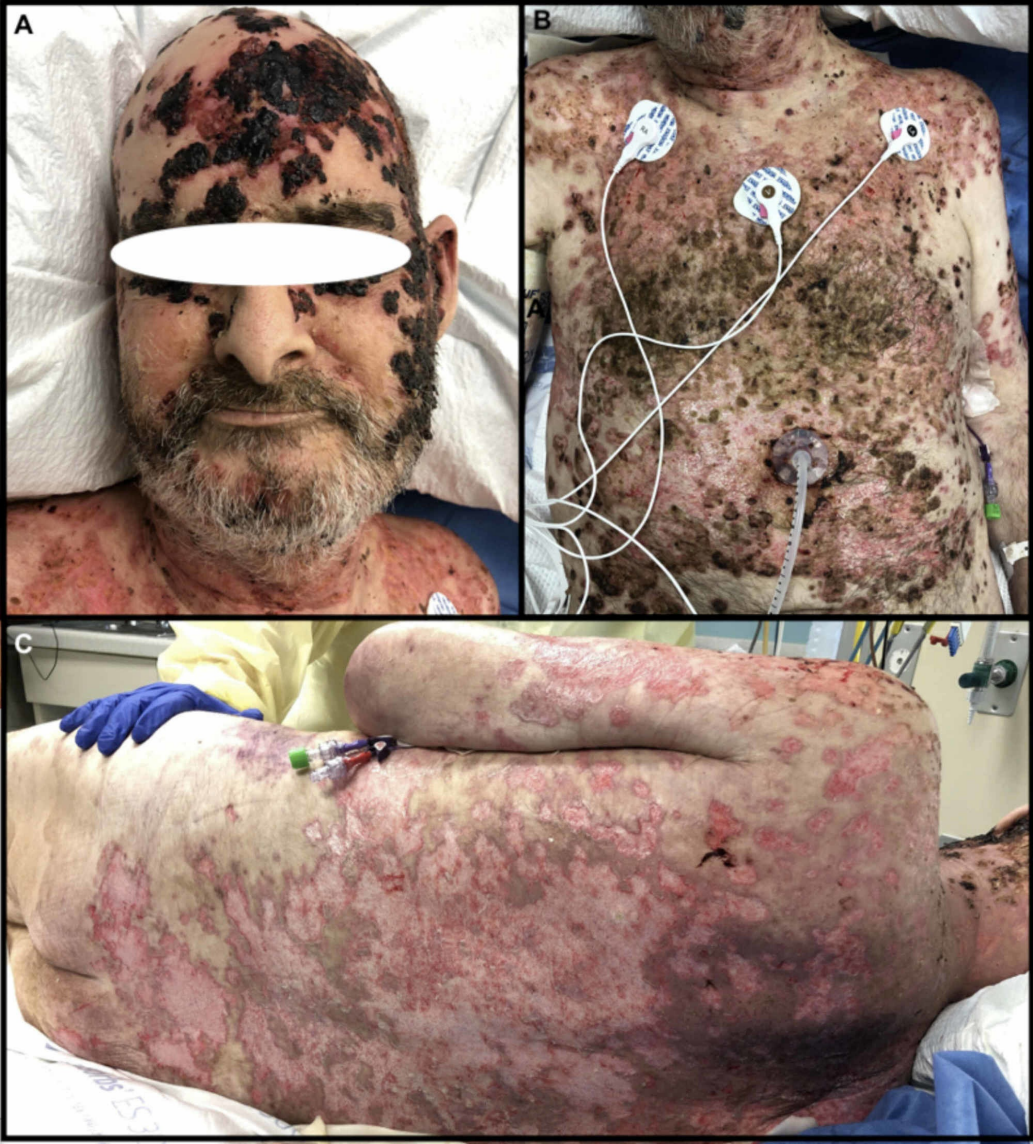
Presentation Upon Admittance to Burn Center. (A – Anterior Head, B – Anterior Torso, C – Posterior Torso)

The on-call dermatologist was consulted, and perilesional punch biopsy from the left lateral trunk was collected for wound culture and expert dermatopathologist histopathological examination. The culture confirmed *Staphylococcus aureus* which suggested possible staphylococcal-scalded-skin-syndrome. However, the dermatopathologist assessed the sample via direct immunofluorescence (IF) and reported heavy linear deposition of IgG and C3 on the cell surface of keratinocytes with subcorneal acantholysis in the epidermis. The patient's blood was also sent for indirect IF and enzyme-linked immunosorbent assay (ELISA) for desmoglein (DSG) 1 and 3 (DSG1 > 100, reference < 18). These findings along with the clinical picture are diagnostic for PF. The patient was discharged with instructions for antibiotic control of the infection. Two weeks later, he re-presented to the ED with a diffuse body rash, swelling, and uncontrollable pain. Workup suggested acute kidney injury (AKI) and methicillin-resistant *Staphylococcus aureus* (MRSA) bacteremia. He was admitted for inpatient management of his bacteremia and PF where he was treated with long-term steroids, a five-day course of intravenous Immunoglobulin (IVIG), and one dose of rituximab, stabilizing his PF. He was discharged with planned treatment of weaning off steroids, monthly IVIG, and a single dose of rituximab every three months. Upon discharge, the patient was also advised to seek continuing follow-up consultations to monitor his pemphigus. After six weeks, he was readmitted to our ED with a worsening weeping, blistering rash, declining mental status, and dyspnea (Figure 2). 

**Figure 3:**
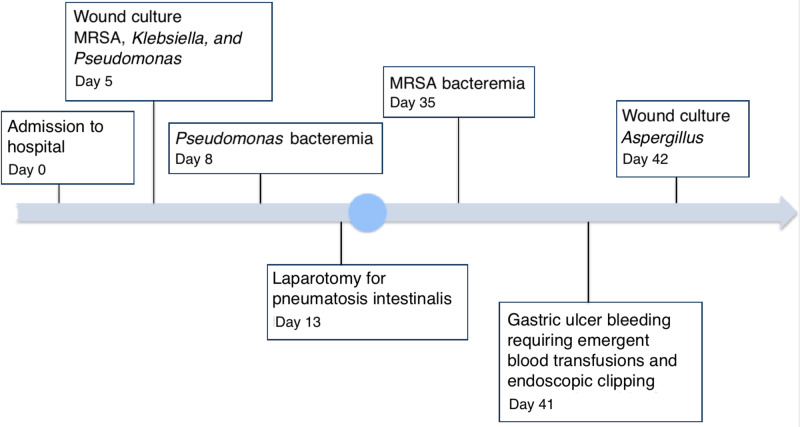
Timeline of events in the burn unit MRSA, methicillin-resistant *Staphylococcus aureus*

Pemphigus wounds are not usually surgically managed, but due to persistent systemic infections the patient required excision, restructuring, and resurfacing for which he was transferred to our burn unit. He developed bacteremia from his wounds and was treated with a fifth-generation cephalosporin which cleared it. However, on rounds he was noticed to have abdominal distention and an X-ray suggested ileus. Given the severe clinical deterioration a CT scan was obtained and it confirmed pneumatosicoli and pneumoperitoneum. He was immediately taken to the operating room where the acute surgical care team performed an emergent exploratory laparotomy demonstrating viable bowel and no intraabdominal pathology as the cause of his decline. His pneumonia was treated with 10 days of ceftolozane/tazobactam. He improved hemodynamically and his PF is under better control with tapered steroids and local wound care. He is scheduled for outpatient IVIG and rituximab treatment. A brief illustrated timeline of the events is shown in Figure 3.

## Discussion

PF is an autoimmune disorder with no cure [8]. While it is not fatal and its symptoms are less life-threatening than other pemphigus subtypes, quality of life is severely reduced, limiting the patient’s ability to carry out daily tasks as well as negatively affecting their psychological state [9-11]. The current gold standard for chronic management of PF is systemic corticosteroids with steroid-sparing immunosuppressants [12].

Treatment of our patient was complicated due to multiple systemic inflammatory insults. However, we adhered to expert dermatologic guidelines with an immunosuppressant regimen which included four courses of IVIG (five days per month), two courses of rituximab (every three months), and high-dose prednisone (60 mg/day) throughout the patient’s hospitalization. Although the patient’s overall skin disease burden improved over time, the complications of AKI and multidrug-resistant bacteremia, including fungemia (*Candida albicans*) and sepsis, frequently happened following administration of IVIG therapy. The patient did not suffer any significant adverse effects from the rituximab treatment and prednisone therapy, which was weaned down to 10 mg/day from 60 mg/day at onset. In light of these observations, it is prudent to maintain adequate hydration, aggressive wound care, and caution for systemic infections because they can be fatal. Surgical wound debridement is non-standard treatment but in order to control fatal infectious complications, we suggest surgical excision is often needed. Fortunately, our patient’s condition improved significantly and his wounds were manageable allowing for discharge to a skilled nursing facility. 

PF is very rare in North America and more prevalent in South America and North Africa with an annual sporadic incidence of less than one per million individuals per year [3,13,14]. The combination of low mortality and low prevalence in the Western world leads to lack of innovation in the development of curative therapy. There are only four ongoing clinical trials testing new drug therapies, one of which is outside of the US. These new targeted therapies include the use of naturally occurring regulatory T cells to potentially replace chronic immunosuppressive therapies, disruption of B-cell receptor signaling, and neonatal Fc receptor inhibition [15]. In addition to new drug therapies being developed, other treatment methods are being utilized outside of the US. For example, particularly in Europe, extracorporeal immunoadsorption is performed to remove the autoantibodies from the blood [16-18]. PF patients are suffering and they need more effective treatment methods that do not carry an increased risk of harm.

## Conclusions

Given its purely superficial penetration, PF has no direct fatal complication and has even been shown to have historically positive outcomes. However, blisters and subsequent wounds are susceptible to dangerous secondary infections. Since there is no acute life threat from PF, few treatments are available for the condition itself. Instead, therapy relies on managing PF and treating subsequent infections. Because PF is an autoimmune disorder it is managed using immunosuppressants. However, these drugs can make the patient susceptible to multiple systemic infections, which can be life-threatening. The development of new treatment methods is crucial for more efficient management of the condition as well as to improve the patients' quality of life.
